# Hepatic de novo lipogenesis is suppressed and fat oxidation is increased by omega-3 fatty acids at the expense of glucose metabolism

**DOI:** 10.1136/bmjdrc-2019-000871

**Published:** 2020-03-17

**Authors:** Charlotte J Green, Camilla Pramfalk, Catriona A Charlton, Pippa J Gunn, Thomas Cornfield, Michael Pavlides, Fredrik Karpe, Leanne Hodson

**Affiliations:** 1 University of Oxford, Oxford, Oxfordshire, UK; 2 Translational Gastroenterology Unit, John Radcliffe Hospital, Oxford, UK; 3 National Institute for Health Research Oxford Biomedical Research Centre, Oxford University Hospitals Foundation Trust, Oxford, UK

**Keywords:** omega-3 fatty acids, de novo lipogenesis, fatty acid oxidation, liver fat

## Abstract

**Objective:**

Increased hepatic de novo lipogenesis (DNL) is suggested to be an underlying cause in the development of nonalcoholic fatty liver disease and/or insulin resistance. It is suggested that omega-3 fatty acids (FA) lower hepatic DNL. We investigated the effects of omega-3 FA supplementation on hepatic DNL and FA oxidation using a combination of human in vivo and in vitro studies.

**Research design and methods:**

Thirty-eight healthy men were randomized to take either an omega-3 supplement (4 g/day eicosapentaenoic acid (EPA)+docosahexaenoic acid (DHA) as ethyl esters) or placebo (4 g/day olive oil) and fasting measurements were made at baseline and 8 weeks. The metabolic effects of omega-3 FAs on intrahepatocellular triacylglycerol (IHTAG) content, hepatic DNL and FA oxidation were investigated using metabolic substrates labeled with stable-isotope tracers. In vitro studies, using a human liver cell-line was undertaken to gain insight into the intrahepatocellular effects of omega-3 FAs.

**Results:**

Fasting plasma TAG concentrations significantly decreased in the omega-3 group and remained unchanged in the placebo group. Eight weeks of omega-3 supplementation significantly decreased IHTAG, fasting and postprandial hepatic DNL while significantly increasing dietary FA oxidation and fasting and postprandial plasma glucose concentrations. In vitro studies supported the in vivo findings of omega-3 FAs (EPA+DHA) decreasing intracellular TAG through a shift in cellular metabolism away from FA esterification toward oxidation.

**Conclusions:**

Omega-3 supplementation had a potent effect on decreasing hepatic DNL and increasing FA oxidation and plasma glucose concentrations. Attenuation of hepatic DNL may be considered advantageous; however, consideration is required as to what the potential excess of nonlipid substrates (eg, glucose) will have on intrahepatic and extrahepatic metabolic pathways.

**Trial registration number:**

NCT01936779.

**Significance of this study**

**What is already known about this subject?**

- Supplementation with the marine-derived omega-3 fatty acids, eicosapentaenoic acid and docosahexaenoic acid, significantly decrease plasma triacylglycerol concentrations and may reduce intrahepatic triacylglycerol content.
- Animal and in vitro cellular studies have shown that omega-3 fatty acids have a hepatocyte-specific effect at the level of gene transcription where they co-ordinately downregulate hepatic lipogenesis and upregulate fatty acid oxidation.

**What are the new findings?**

- Supplementation with omega-3 fatty acids in humans downregulated fasting and postprandial hepatic de novo lipogenesis and increased postprandial fatty acid oxidation.
- We found fasting and postprandial plasma glucose concentrations increased after supplementation with omega-3 fatty acids.

**How might these results change the focus of research or clinical practice?**

- When hepatic de novo lipogenesis is attenuated, consideration needs to be given to the impact excess nonlipid substrates may have on other metabolic pathways.
- Understanding how omega-3 fatty acid supplementation may affect hepatic glucose metabolism in humans in vivo remains to be determined.
- Clarifying the impact background diet has on metabolic pathways when humans are supplemented with omega-3 fatty acids.

## Introduction

Nonalcoholic fatty liver disease (NAFLD), defined as excess intrahepatocellular triacylglycerol (IHTAG) accumulation due to nonalcoholic causes, is a complication in individuals who are obese and/or have type 2 diabetes (T2D).^1^ Increased hepatic de novo lipogenesis (DNL) is often suggested to be an underlying cause in the development of NAFLD and/or insulin resistance.^2^ Observational studies have reported fasting hepatic DNL to be higher in individuals with NAFLD compared with those without.^3 4^ It has been suggested that changes in plasma TAG concentrations are proportional to the amount of hepatic DNL.^5^

Supplementation with the marine-derived omega-3 fatty acids (FAs), eicosapentaenoic acid (EPA) and docosahexaenoic acid (DHA), in doses of ≥3 g/day significantly decrease plasma TAG concentrations^6^ and can reduce IHTAG content.^7^ In vitro cellular and animal models have proposed the mechanisms by which this is achieved include downregulation of lipogenic and upregulation of β-oxidation pathways via hepatic transcription factors.^7^ In-line with omega-3 FAs upregulating β-oxidation, some,^8 9^ but not all,^10^ have reported an increase in fat oxidation, when assessed by indirect calorimetry. However, few have assessed the effect of omega-3 FAs on fasting and postprandial hepatic DNL and FA oxidation, simultaneously, in vivo, in humans.

The effects of omega-3 supplementation on markers of glycemia are inconsistent. For example, Logan *et al*^8^ reported no change in older, overweight/obese females taking 3 g EPA+DHA for 12 weeks, while others report increased fasting plasma glucose concentrations in individuals with T2D taking 20 mL fish oil for 9 weeks^9^ or 5 g EPA+DHA for 24 weeks^11^; in one case, a decrease was reported in overweight/obese adults with impaired glucose tolerance taking 3 g EPA+DHA for 18 months.^12^ The disparity in findings is likely related to the dose and duration of omega-3 supplementation along with the clinical status of the individuals studied. In a pilot study, we previously found supplementation with 4 g/day EPA+DHA for 15–18 months in individuals with NAFLD did not alter fasting plasma glucose concentrations, but decreased fasting hepatic DNL and increased plasma 3-hydroxybutryate (3OHB) concentrations.^13^ Therefore, the aim of this work was to extend our previous observations and investigate the effect of 8 weeks of EPA+DHA (omega-3) supplementation on fasting and postprandial hepatic DNL and FA oxidation using a combination of human in vivo and in vitro models and stable-isotope tracer methodology.

## Research design and methods

### In vivo human studies

Healthy male participants, with fasting plasma TAG concentrations 1.5 mmol/L or greater were recruited from the Oxford BioBank (OBB).^14^ All volunteers were nondiabetic and free from any known disease, had a body mass index <35 kg/m^2^, were not taking medication known to affect lipid or glucose metabolism, did not smoke, did not consume alcohol above recommended limits^15^ and were not taking any supplements enriched with omega-3 FAs.

#### Study design, randomization and supplementation

A total of 57 men (aged 35–55 years) were screened with 47 meeting the inclusion criteria. Participants were randomly assigned to consume either omega-3 FA ethyl esters (4 g/day, n=24) or placebo (4 g/day, n=23) and were studied before and 8 weeks after supplementation (online supplementary figure 1). Participants randomized to the omega-3 FA group consumed 4×1 g capsules/day with each capsule containing 460 mg EPA ethyl ester and 380 mg DHA ethyl ester (a total of 1.84 g EPA+1.52 g DHA/day (medicinal product name Omacor/Lovaza)). Participants randomized to the placebo group consumed 4×1 g capsules/day with each capsule containing olive oil (FA composition oleic acid ~67%, palmitic acid ~15%, linoleic acid ~15% and stearic acid ~2%, alpha linolenic acid ~1%). This was an open-label study and the dose of 4 g/day was based on previous studies^16 17^ and olive oil was chosen as a placebo as oleic acid is a commonly consumed FA due to its ubiquitous nature in foods^18^ and has been used previously.^10 16 17^ Both the omega-3 and placebo were administrated as 1 g, soft red-brown, gelatin shelled capsules and participants were encouraged to take the supplement with the first meal of the day. Supplements (omega-3 and placebo) were provided by Pronova BioPharma (now part of BASF) (Pronova Bio-Pharma ASA, Lysaker, Norway).

10.1136/bmjdrc-2019-000871.supp1 Supplementary data

We determined the 90th percentile of plasma TAG concentrations for males in the OBB was 2.2 mmol/L (SD of 0.5 mmol/L). Based on the work of Chan *et al*,^19 20^ who reported a 25% decrease in plasma TAG, in males after 6 weeks of omega-3 FA supplementation (4 g/day EPA+DHA), we predicted a 20% decrease in plasma TAG after 8 weeks supplementation with 4 g/day of omega-3 FA. The number of individuals required to detect a 20% decrease in plasma TAG, with a power of 0.80 and α of 0.05, was n=21. The supplementation period of 8 weeks was based on the work of Cussons *et al.*^17^

Whole body composition and fat distribution were measured using dual-energy X-ray absorptiometry^21^ at the baseline visit.

#### Placebo group

Of the 23 individuals randomized, 4 did not complete the study due to changes in personal and/or working circumstances, therefore giving complete data for 19 individuals (online supplementary figure 1). Participants came in to the Clinical Research Unit after an overnight fast for fasting blood samples, at the beginning and 8 weeks after supplementation with placebo capsules.

#### Omega-3 supplementation group

Of the 24 participants randomized, 3 did not complete the study due to changes in personal and/or working circumstances leaving 21 participants for whom we had fasting data. As we wanted to investigate the effect of omega-3 FAs on hepatic DNL and FA partitioning, only participants in this group had IHTAG measured and underwent postprandial study days. From this, we have complete data for 19 individuals as two individuals did not complete the second postprandial study day due to difficulties in obtaining blood samples over the course of the study day (online supplementary figure 1).

#### Compliance

To assess compliance of participants to taking the supplements erythrocyte FA composition was assessed^18^ at baseline and after 8 weeks as described.^22^

#### Measurement of IHTAG content (omega-3 group only)

IHTAG content was measured using proton magnetic resonance spectroscopy within 1 week of the metabolic study day, at baseline and after 8 weeks of omega-3 FA supplementation as described.^23^

#### Metabolic study day (omega-3 group only)

Prior to the study day, subjects were asked to avoid foods naturally enriched in ^13^C (eg, cornflakes, popcorn, foods made with corn starch and so on), alcohol and strenuous exercise. The evening prior to the study day, subjects consumed deuterated water (^2^H_2_O) (3 g/kg body water) and continued to consume ^2^H_2_O during the course of the study day for the measurement of fasting and postprandial hepatic DNL.^24^

On the study day, after an overnight fast and consumption of ^2^H_2_O, subjects came to the Clinical Research Unit and a cannula was inserted into an antecubital vein and baseline (Time 0) blood and breathe samples taken. Participants were then fed a mixed test meal, consisting of 40 g Rice Krispies (Kelloggs, Manchester, UK), 200 g skimmed milk and a chocolate drink containing 40 g olive oil (40 g fat, 40 g carbohydrate). Two hundred mg of [U^13^C]palmitic acid was emulsified with the chocolate drink to trace the fate of the dietary FAs. Repeated blood and breath samples were taken over the course of the study period.

Indirect calorimetry was performed at Time 0 (fasting) and then 120 min after consumption of the test meal using a GEM calorimeter (GEMNutrition, Daresbury, Cheshire, UK) to determine whole-body CO_2_ production, whole-body respiratory exchange ratio and substrate utilization rates.

#### Analytical methods

Whole blood was collected into heparinized syringes (Sarstedt, Leicester, UK) and plasma was rapidly separated by centrifugation at 4 ºC for the measurement of plasma metabolite and insulin concentrations as described.^25^ Separation of the chylomicron fraction ((Svedberg flotation rate, S_f_)>400) and the very low-density lipoprotein (VLDL)-rich fraction (S_f_20-400) were made by sequential flotation using density gradient ultracentrifugation and anti-ApoB100 immunoabsorption capture to obtain a fraction completely devoid of apoB48 and hereafter called VLDL, as described.^25^

Samples were taken at Time 0 (baseline) and then 30, 60, 90, 120, 180, 240, 300 and 360 min after the consumption of the test meal for the measurement of plasma glucose, insulin, TAG, nonesterified fatty acids (NEFA), 3OHB and at 0, 15, 30, 60, 90, 120, 180, 240, 300 and 360 min for the analysis of chylomicron-TAG, TAG-rich lipoproteins-TAG and 0, 180, 240, 300 and 360 min for the analysis of VLDL-TAG. Breath samples were collected at 0, 60, 90, 120, 180, 240, 300 and 360 min into EXETAINER tubes (Labco, High Wycombe, Bucks, UK) for measurement of expired ^13^CO_2_ enrichment.

#### FA and isotopic enrichment

To determine the specific FA composition and isotopic enrichment, total lipids were extracted from plasma and lipoproteins. FA methyl esters were prepared from plasma NEFA, and TAG fractions, along with erythrocyte total phospholipids as described.^22^ The FA compositions (µmol/100 µmol total FA) in these fractions were determined by gas chromatography (GC) and in plasma NEFA and TAG fractions palmitate concentrations were calculated as described.^25^

[U^13^C]palmitate enrichments were measured in plasma NEFA, TAG, S_f_ >400 (chylomicron-TAG), S_f_ 20–400-TAG and VLDL-TAG FA methyl ester derivatives using a Delta Plus XP GC-combustion isotope ratio mass spectrometer (Thermo electron, Bremen, Germany).^26^ The tracer-to-tracee ratio (TTR) of a baseline measurement (before administration of [U^13^C]palmitate) was subtracted from the TTR of each sample to account for natural abundance and then multiplied by the corresponding palmitate concentrations to give plasma and lipoprotein tracer concentrations.^25^

We estimated dietary FA oxidation at the whole-body and hepatic level. By collecting breath samples relative rate of whole-body meal-derived FA oxidation were calculated as described.^26^ The liver is the only organ to produce urea in significant amounts and CO_2_ produced from hepatic FA oxidation is used in urea synthesis.^27^ We used stable-isotope methodologies to measure ^13^CO_2_/^12^CO_2_ ratios liberated from plasma urea, based on the method of Kloppenburg *et al*^28^ to assess whether recently ingested dietary FAs were undergoing complete intrahepatic oxidation. To allow for sequestration of label into the bicarbonate pool a dietary acetate recovery factor of 51% was applied.^29^ Hepatic CO_2_ production was not measured directly, but calculated using reported splanchnic respiratory quotients.^30^

Fasting and postprandial hepatic DNL was assessed based on the incorporation of deuterium from ^2^H_2_O in plasma water (Finnigan Gas-Bench-II; Thermo Fisher Scientific, Loughborough, UK) into VLDL-TG palmitate using GC–mass spectrometry with monitoring ions with mass-to-charge ratios (m/z) of 270 (M+0) and 271 (M+1) and percentage DNL calculated as described.^31^

### In vitro cellular studies

All reagents were obtained from Life Technologies (Paisley, Scotland) unless otherwise stated. Fetal bovine serum (FBS) was purchased from Seralab (West Sussex, UK). FAs were purchased from Cambridge Bioscience (Cambridge, UK). FA free bovine serum albumin (BSA), was purchased from SIGMA Aldrich (UK). KAPA probe fast mix was purchased from KAPA Biosystems (London, UK).

#### Cell culture

Huh7 cells were grown as described.^32^ Confluent cells were cultured in DMEM (11 mM)+Glutamax, 10% FBS, 1× nonessential amino acids, 1% penicillin-streptomycin, for 48 hours prior to FA treatment. Cells were first treated with 100 µM FA (containing oleic (O, 45%), palmitic (P, 30%) and linoleic (L, 25%) acid (OPL) conjugated to 0.25% FA-free BSA giving a FA:BSA molar ratio of 3:1 for 24 hours. Next, the cells were treated with 200 µM FA (FA:BSA molar ratios of 3:1) mixtures for 48 hours that consisted of either OPL alone (90 µM oleic, 60 µM palmitic and 50 µM linoleic acid or OPL+EPA+DHA (49.5 µM oleic, 33 µM palmitic, 27.5 µM linoleic+55 µM EPA+45 µM DHA). EPA and DHA stocks were stored under nitrogen and cells and media were collected on ice for TAG measurement, which was quantified as described.^32^ RNA extraction was carried out using QIAGEN RNeasy Mini Kit according to manufacturer’s instructions.

#### FA and isotopic enrichment

To determine the effect of EPA+DHA on the contribution of DNL-derived FA to intracellular TAG, 50% (5.5mM) of media glucose was labeled with [U^13^C] for 48 hours. Cells were collected for analysis and the contribution of glucose-derived FA in intracellular TAG determined using GC-mass spectrometry as described.^33^

To trace the oxidation of exogenous FA, Huh7 cells were treated with OPL and OPL+EPA+DHA where palmitate was labeled (100% D_31_ palmitate) for 48 hours. As a marker of FA oxidation, the appearance of ^2^H_2_O (derived from [D_31_]palmitate) in media was measured, using a Finnigan GasBench-II (ThermoFisher Scientific, UK).^34^

#### Quantitative real time PCR

First strand cDNA was synthesized from 0.5 µg total RNA using a High Capacity Reverse Transcription kit. RT-PCR reactions were run on an Applied Biosystems 7900HT machine. Two housekeeping genes were used: tyrosine 3-monooxygenase/tryptophan 5-monooxygenase activation protein zeta and beta-2-microglobulin.

### Calculations and statistical methods

Homeostatic model assessment of insulin resistance (HOMA-IR) was calculated.^35^ Data were analyzed using SPSS for Windows V.22 (SPSS, Chertsey, UK). All data are presented as means±SEM unless otherwise stated. Areas under the curve (AUCs) were calculated by the trapezoid method. AUCs have been divided by the relevant time period to give time-averaged values. All data sets were tested for normality according to the Shapiro-Wilk test. Comparisons between the placebo and omega-3 groups were made using an independent t-test or Mann Whitney U tests for nonparametric data. For comparisons within the groups before and after supplementation were made using a students paired t-test or the nonparametric equivalent. Postprandial data were compared using repeated measures analysis of variance, with time and treatment as factors to investigate the change within the omega-3 FA group over time for specific metabolites. Bonferroni posthoc analysis was performed where appropriate to adjust for multiple comparisons. Associations between variables were carried out using Spearman’s rank correlation coefficient. Statistical significance was set at p<0.05.

For in vitro cell studies, data were analyzed using GraphPad Prism 7 software using an independent t-test or Mann Whitney U test for nonparametric data (GraphPad software, La Jolla, USA).

## Results

### In vivo human study

#### Participant characteristics

Thirty-eight subjects (19 placebo and 19 omega-3) completed the study. There was no difference in age, weight, body mass index or waist circumference between or within the groups at baseline and after 8 weeks (table 1). There was no difference between the groups in body composition, which was assessed at the baseline visit with total fat, lean and visceral fat masses being 26.3±1.5 kg vs 27.6±1.9 kg, 60.8±1.6 kg vs 60.5±1.6 kg and 1.7±0.1 kg vs 1.8±0.2 kg, placebo vs omega-3, respectively. Between baseline and 8 weeks fasting plasma TAG and ALT concentrations significantly (p<0.01) decreased, while plasma glucose significantly (p<0.05) increased in the omega-3 group while between the groups the only significant (p<0.05) difference was the change in plasma TAG and ALT concentrations (table 1).

**Table 1:** Characteristics of study participants at baseline and 8 weeks

	Placebo (n=19)			Omega-3 (n=19)		
	Baseline	8 weeks	Change (%)	Baseline	8 weeks	Change (%)
Age (years)	45 (33–52)			45 (27–52)		
Weight (kg)	91 (74–117)	91 (74–115)	0.0±0.4	91 (69–115)	91 (68–115)	0.1±0.5
BMI (kg/m^2^)	27.6 (22.0–35.1)	27.8 (21.6–34.6)	0.0±0.4	28.9 (24.6–34.6)	28.7 (24.1–34.8)	0.1±0.5
Waist (cm)	100 (86–116)	99 (87–114)	0.9±0.4	99 (94–121)	99 (92–122)	−1.0±0.7
Hip (cm)	103 (93–113)	103 (94–113)	0.0±0.3	104 (94–121)	104 (92–123)	0.2±0.6
HOMA-IR	3.2±0.3	3.3±0.3	2.9±6.1	3.5±0.3	3.5±0.2	8.8±6.2
*Fasting plasma biochemical parameters*						
Glucose (mmol/L)	5.4±0.1	5.5±0.1	1.9±2.0	5.5±0.1	5.7±0.1*	4.2±2.0
Insulin (mU/L)	13.4±1.0	13.2±1.0	0.3±5.0	14.1±1.1	14.1±0.8	3.9±5.4
NEFA (µmol/L)	388±30	389±33	6.6±12.0	371±30	346±30	−2.7±7.4
Total cholesterol (mmol/L)	5.6±0.2	5.5±0.2	−0.2±5.5	5.7±0.2	5.6±0.2	−0.4±3.7
HDL cholesterol (mmol/L)	1.23±0.06	1.24±0.05	0.5±2.4	1.02±0.04	1.03±0.03	3.5±4.2
Non-HDL cholesterol (mmol/L)	4.6±0.2	4.2±0.2	−3.3±7.7	4.7±0.2	4.5±0.2	−1.1±3.4
TAG (mmol/L)	2.2±0.2	2.2±0.2	2.8±7.7	2.2±0.2	1.7±0.2**	−18.1±6.7†
3OHB (µmol/L)	51±9	61±8	39±32	54±9	50±6	4±9
ALT (IU/L)	32±2	32±2	3.9±6.5	36±4	26±2**	−16.6±6.6†
*Erythrocyte fatty acids(mol%)*						
EPA (20:5 n-3)	1.1±0.1	1.1±0.1	−1.4±3.4	1.0±0.1	2.9±0.2***	224±32‡‡‡
DPA (22:5 n-3)	5.7±0.2	5.6±0.2	−0.8±1.3	5.7±0.1	6.0±0.2**	4.7±1.6†
DHA (22:6 n-3)	8.3±0.3	8.2±0.3	−1.8±1.2	8.4±0.2	9.5±0.3***	12.4±1.9‡‡‡

Data expressed as median (min–max) or mean±SEM. Change (%) expressed as mean±SEM.

*P<0.05, **p<0.01, ***p<0.001 baseline vs 8 weeks within the group.

‡P<0.05, ‡‡p<0.01, ‡‡‡p<0.001% change in placebo vs omega-3 group.

ALT, alanine transaminase; BMI, body mass index; DHA, docosahexaenoic acid; DPA, docosapentaenoic acid; EPA, eicosapentaenoic acid; HDL, high density lipoprotein; HOMA-IR, homeostatic model assessment of insulin resistance; NEFA, nonesterified fatty acids; 3OHB, 3-hydroxybutyrate; TAG, triacylglycerol.

#### Compliance

Compared with the placebo group, there was a significant (p<0.05) increase in erythrocyte EPA, docosapentaenoic acid (DPA) and DHA in the omega-3 group between baseline and 8 weeks (table 1). Within the omega-3 group, there was a graded response with the relative change in erythrocyte EPA content ranging from 69% to 537% and the relative change in erythrocyte DHA content was from a small decrease of −6% to an increase of 24%. There was a strong inverse association between baseline levels of erythrocyte EPA and the per cent change in EPA achieved (r_s_=−0.73, p<0.001) while there was no association between baseline erythrocyte DHA levels and per cent change in DHA (r_s_=−0.28, p=NS).

#### The effect of omega-3 supplementation on IHTAG and plasma postprandial metabolites (omega-3 group only)

**Figure 1:**
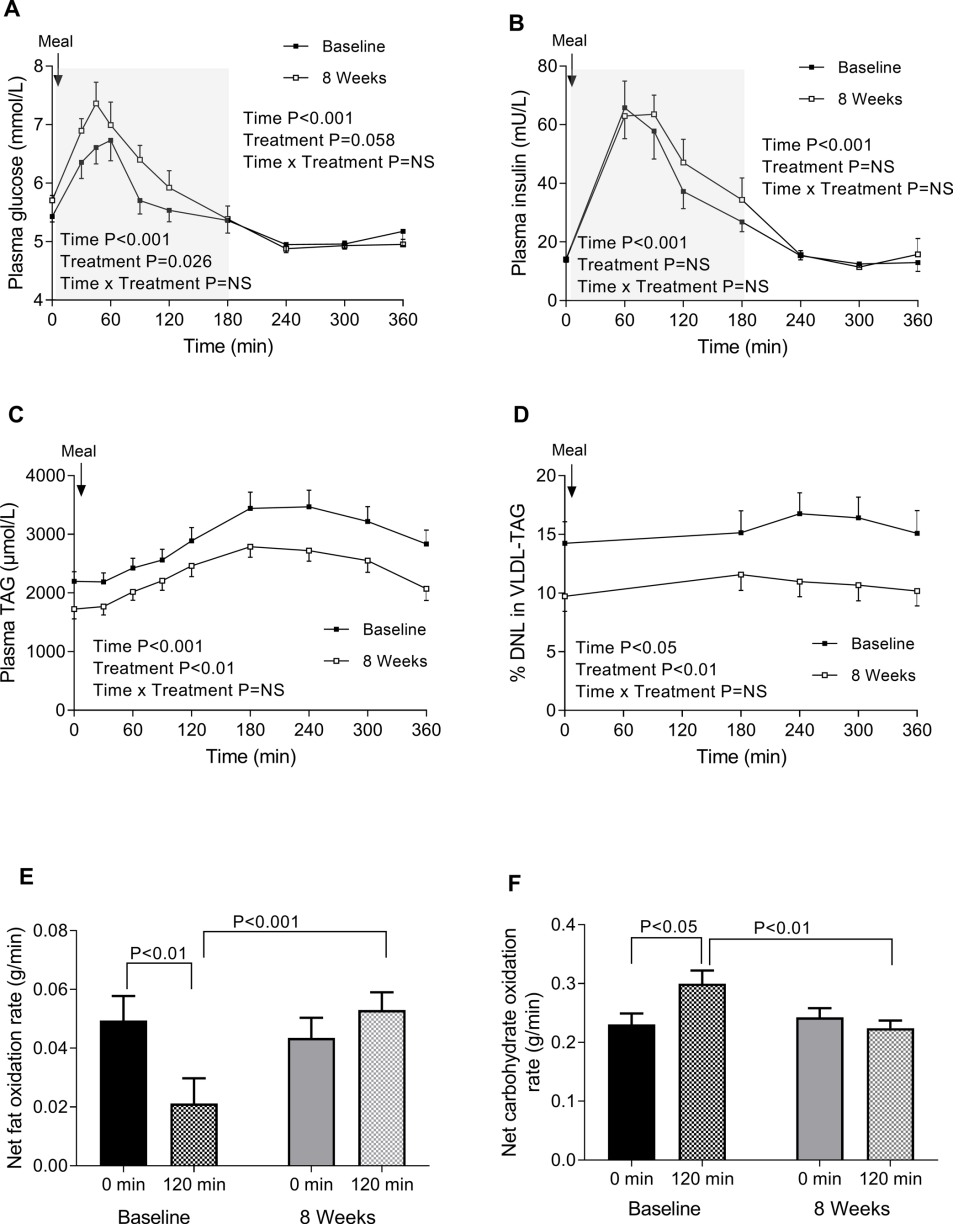
The effect of omega-3 FA supplement (omega-3 FA group only) at baseline and 8 weeks on: (A) plasma glucose; (B) plasma insulin; (C) plasma TAG; (D) per cent of DNL-derived FAs in VLDL-TAG; (E) net fat oxidation (baseline and 120 min postprandial) and (F) net carbohydrate oxidation (baseline and 120 min postprandial). Data are presented as means±SEM. DNL, de novo lipogenesis; FA, fatty acid; VLDL, very low-density lipoprotein; TAG, triacylglycerol.

Eight weeks of omega-3 supplementation significantly (p<0.05) decreased IHTAG by 19% (table 2). The increase (p<0.05) in fasting plasma glucose concentrations at 8 weeks was maintained after consumption of the mixed test meal (time-averaged 180 min AUC, 5.9±0.2 mmol/L vs 6.3±0.2 mmol/L, baseline vs 8 weeks, p<0.05) and when considered over 360 min, there was a tendency (p=0.058) for higher fasting plasma glucose concentrations at 8 weeks compared with baseline (figure 1A). Omega-3 supplementation had no effect on postprandial plasma insulin (figure 1B) nor systemic NEFA (table 2) concentrations. The significant (p<0.01) decrease in fasting plasma TAG concentrations after omega-3 supplementation were maintained over the course of the postprandial period (figure 1C), which can in part be explained by a significant decrease in fasting and postprandial VLDL-TAG concentrations (table 2). Although there was no change in VLDL-apolipoprotein (Apo)B concentrations with omega-3 supplementation, there was a significant decrease in the VLDL-TAG to VLDL-ApoB ratio, suggesting a decrease in particle size due to lower TAG content, rather than a decrease in particle number (table 2). We correlated the changes from baseline to 8 weeks for IHTAG and VLDL-TAG to VLDL-ApoB ratio and found a positive association (r_s_=0.54, p<0.05) (online supplementary figure 2). There was no change in postprandial plasma 3OHB concentrations after omega-3 supplementation (table 2).

10.1136/bmjdrc-2019-000871.supp2 Supplementary data

**Table 2:** IHTAG content and fasting and postprandial biochemical characteristics of participants (n=19) taking omega-3 FA

	Baseline	8 weeks
IHTAG (%)	7.8±1.6	6.3±1.3*
*Fasting plasma biochemical parameters*		
VLDL-TAG (µmol/L)	1335±57	1069±97*
VLDL-ApoB (mg/dL)	3.5±0.5	4.6±0.7
VLDL-TAG/VLDL-ApoB†	34 791±6600	17 657±2945*
*Postprandial plasma biochemical parameters (AUC, time-averaged)*		
Chylomicron-TAG (µmol/L)	564±70	449±52
VLDL-TAG (µmol/L)	1546±57	1330±79*
NEFA (µmol/L)	89±21	80±18
3OHB (µmol/L)	81.5±11.3	87.3±11.6
*Postprandial*^*13*^*C-labeled plasma biochemical parameters (AUC, time-averaged)*		
^13^C plasma TAG-palmitate (µmol/L)	3.9±0.4	3.3±0.3
^13^C chylomicron-TAG palmitate (µmol/L)	2.9±0.3	2.0±0.2*
^13^C NEFA-palmitate (µmol/L)	0.43±0.04	0.45±0.03
^13^C VLDL-TAG palmitate (µmol/L)	0.87±0.07	0.79±0.05
Whole-body ^13^CO_2_ (µmol/min)	2.8±0.2	3.2±0.2*
Hepatic ^13^CO_2_ (µmol/min)	0.91±0.07	1.0±0.06*
Respiratory exchange ratio	0.96±0.02	0.89±0.01**

Data expressed as mean±SEM.

*P<0.05, **p<0.01 baseline vs 8 weeks.

†Molar ratio.

AUC, areas under the curve; IHTAG, intrahepatic triacylglycerol; NEFA, nonesterified fatty acids; 3OHB, 3-hydroxybutyrate; TAG, triacylglycerol; VLDL, very low-density lipoprotein.

Supplementation with omega-3 FA resulted in a 30% decrease (p<0.05) in fasting and postprandial hepatic DNL (figure 1D). There was no association between changes in DNL (fasting or postprandial) with change in IHTAG or plasma TAG concentrations (online supplementary figure 2).

#### The effects of omega-3 supplementation on ^13^C dietary FA metabolism (omega-3 group only)

There was a significant (p<0.05) decrease in the appearance of ^13^C (from dietary fat) into chylomicron-TAG but not VLDL-TAG while whole-body and hepatic ^13^CO_2_ production significantly (p<0.05) increased after omega-3 supplementation (table 2). There was a significant increase in the respiratory exchange ratio (RER) between the fasting and postprandial measurement at baseline (going from 0.89±0.02 to 0.96±0.02, p<0.01), which was not evident at 8 weeks (going from 0.90±0.01 to 0.89±0.01, p=NS). These data suggest that the fraction of ingested carbohydrate that was then synthesized into new fat via DNL would be minimal. In line with this the whole-body and hepatic CO_2_ data, along with the significant decrease in postprandial RER between baseline and 8 weeks, supports a shift toward FA oxidation (table 2). We calculated net substrate oxidation and found an increase (p<0.001) in the postprandial net fat oxidation rate (g/min) (figure 1E) and a concomitant decrease (p<0.01) in postprandial net carbohydrate oxidation rate (g/min) (figure 1F) after omega-3 supplementation.

### In vitro cellular data

To further investigate the mechanisms behind the effects observed in vivo in humans we undertook in vitro cellular studies using Huh7 cells and a FA mixture of OPL.

#### Effect of omega-3 on intracellular TAG content

**Figure 2:**
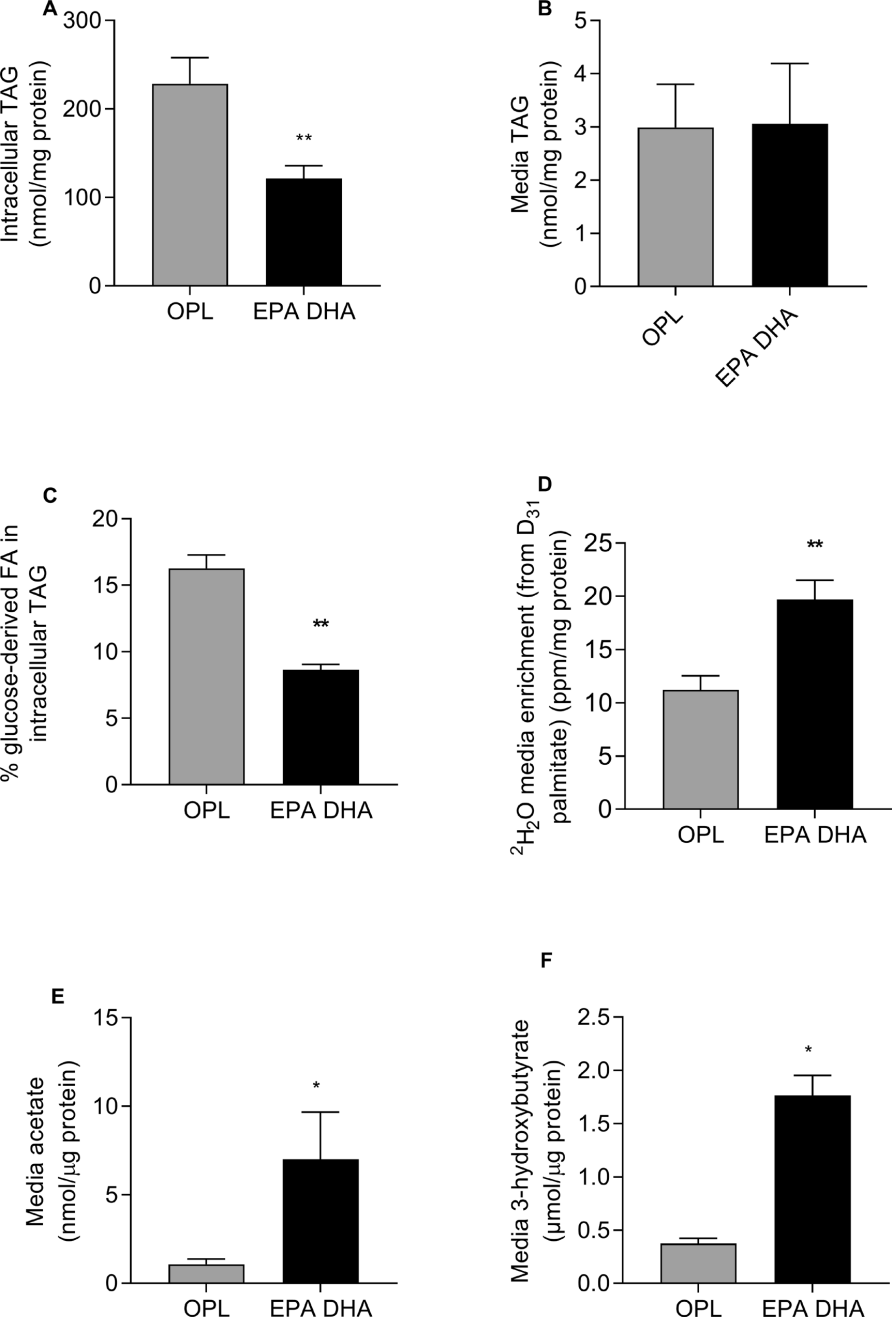
Huh7 cells (n=6 per group) were treated with 200 µM FAs for 48 hours. Cells and media were collected and (A) intracellular TAG content and (B) media TAG content measured. Cells were treated with 200 µM FAs for 48 hours with ^13^C glucose or D_31_ palmitate added to the culture media and cells and media were collected for the measurement of the effect of EPA+DHA on: (C) the relative contribution (%) of glucose-derived DNL FAs to intracellular TAG (n=4) and (D) FA oxidation as measured by ^2^H_2_O media enrichment (from D_31_palmitate, corrected for tracer enrichment) (ppm/mg protein) in cellular media (n=6). Data are presented as means±SEM. ^*^P<0.05; ^**^p<0.01; p<0.001 vs OPL. DNL, de novo lipogenesis; EPA DHA, OPL+EPA+DHA; FA, fatty acid; OPL, oleate, palmitate, linoleate; TAG, triacylglycerol.

The addition of omega-3 (EPA+DHA) to OPL resulted in a significant decrease (p<0.01) in intracellular TAG content, with no change in media TAG concentration (figure 2A, B).

#### Effect of omega-3 on FA synthesis and oxidation

We assessed the effect of OPL and EPA+DHA with OPL only on intracellular DNL using [U^13^C]glucose and found a significant reduction (p<0.01) in the proportion of intracellular DNL (as assessed by the incorporation of ^13^C from glucose into 16:0 ([^13^C]16:0) when omega-3 (EPA+DHA) were present (figure 2C). We assessed the effects of EPA+DHA on FA oxidation by culturing cells in OPL (where P was [D_31_]16:0 and looking for the appearance of ^2^H in media water) with and without EPA+DHA and found a significant (p<0.01) increase in media ^2^H_2_O enrichment when cells were cultured in EPA+DHA compared with cells cultured in OPL alone (figure 2D). FAs may also undergo peroxisomal oxidation; therefore, we assessed the change in media acetate concentrations as a marker of peroxisomal FA oxidation and found a threefold increase (p<0.01) in the media of cells cultured with EPA+DHA compared with cells cultured in OPL alone (figure 2E). We observed a significant (p<0.05) increase in the concentration of 3OHB in media, in cells cultured in EPA+DHA compared with OPL alone (figure 2F).

We measured the relative mRNA expression of genes involved in lipogenic and oxidation pathways in Huh7 cells that had been cultured in OPL and OPL+EPA+DHA. Culturing cells with EPA+DHA resulted in significant decreases in expression of some, but not all genes. The presence of EPA+DHA significantly decreased *FASN, DGAT2,* and *SCD* mRNA levels compared with OPL alone (online supplementary table 1). In contrast to our finding of increased FA oxidation when using stable-isotopes, there was a significant decrease in the *CPT1A* mRNA levels with EPA+DHA compared with OPL alone (online supplementary table 1). The data for the expression of *ACACB* are not presented as the mRNA levels in our cell models were too low to reliably quantify.

10.1136/bmjdrc-2019-000871.supp3 Supplementary data

## Discussion

It is often suggested that increased hepatic DNL is an underlying cause of NAFLD and/or insulin resistance,^2^ the latter of which at the level of the liver, leads to continued gluconeogenesis and accelerated DNL.^36^ Findings from animal and in vitro work show omega-3 FAs have a hepatocyte-specific effect by downregulating the transcription of genes in the lipogenic pathway.^7^ If omega-3 FAs attenuate hepatic DNL in vivo in humans, then this may, in part, explain the hypo-TAG and/or IHTAG lowering effect observed with omega-3 supplementation. Findings for the effect omega-3 FAs have on markers of glycemia and FA oxidation are inconsistent. Therefore, we used a combination of human in vivo and in vitro cellular studies, along with stable-isotope methodology, to investigate the effect of 8 weeks supplementation with omega-3 FAs (EPA+DHA) on fasting and postprandial hepatic DNL and FA oxidation. In line with previous work, we found significant decreases in fasting and postprandial plasma TAG concentrations and IHTAG content.^17^ Plasma ALT has been reported to be positively associated with IHTAG^37^ and the observed decrease in plasma ALT levels after supplementation with omega-3 FA is consistent with a reduction in IHTAG, although a correlation between ALT and IHTAG is not always observed in studies where liver fat has decreased.^7^ In addition, we observed significant decreases in fasting and postprandial hepatic DNL and significant increases in dietary FA oxidation and fasting and postprandial plasma glucose concentrations. Moreover, a striking result in the current study was the reversal of a set of canonical metabolic responses to a mixed meal. At baseline, we observed the usual response to a mixed meal, with the suppression of fat oxidation, to preserve dietary FAs for storage, with dietary carbohydrate being utilized instead. After 8 weeks of omega-3 FA supplementation however, the responses to a mixed meal were remarkably different with fat oxidation significantly increasing and carbohydrate utilization significantly decreasing.

### Omega-3 supplementation decreases hepatic DNL

Although it is often suggested that omega-3 FAs may lower hepatic DNL, this has not been adequately assessed in vivo in humans. In a previous pilot study of patients with NAFLD, we observed that long-term (15–18 months) supplementation with omega-3 FAs decreased fasting hepatic DNL.^13^ In the present study, we observed significant decreases in fasting and postprandial hepatic DNL after 8 weeks of omega-3 supplementation. The lack of response in postprandial DNL observed in the current study is notable and extends our previous observations. Hepatic DNL typically increases after consumption of a mixed meal as within the liver, insulin activates the transcription factor sterol regulatory element-binding protein 1 c (SREBP-1c) which enhances the transcription of genes required for FA and TAG synthesis.^36 38^ Work by McGarry *et al*^39^ demonstrated that malonyl-CoA, an intermediate in the DNL pathway, was a potent inhibitor of carnitine-palmitoyl-transferase 1. Thus, it is plausible that the physiological importance of the DNL pathway is its contribution to the regulation of FA oxidation via this mechanism, rather than its quantitative contribution to FA supply.

By assessing the effect of omega-3 FAs on glucose-derived DNL in vitro in a relevant hepatocyte model (by using [U^13^C]glucose), we found the presence of omega-3 (EPA+DHA) attenuated the relative contribution of glucose-derived FAs to intracellular TAG. We also noted the mRNA levels of some, but not all, genes involved in the DNL pathway were attenuated. Rodent work has previously suggested that omega-3 FA supplementation leads to suppression of SREBP1-c when animals are fed a high carbohydrate/glucose diet for 7 days or 5 months.^40 41^ However, Xu *et al*^41^ noted that after one meal, despite the nuclear protein content of SREBP-1c being decreased, the amount of membrane-anchored precursor SREBP-1c and the abundance of SREBP-1c mRNA were not reduced. Our in vitro model did not recapitulate a lipogenic model that has been used in previous work, where a mixture of glucose and fructose, with no FAs, is present in the culture media.^42^ Rather, we cultured the cells in a mixture of nutritional substrates including a physiological mix of FAs and 11 mM glucose and this may, in part, explain the variability in response in the mRNA levels of genes involved in the DNL pathway.

As changes in plasma TAG concentrations may be proportional to the amount of hepatic DNL,^5^ it is plausible the decrease in DNL observed here played a role in the observed decreases in VLDL-TAG concentrations, and IHTAG content, through shifting cellular metabolism away from esterification of FA toward oxidation.^43^ Although some have reported fasting hepatic DNL to be correlated with IHTAG content and VLDL-TAG secretion rate,^4^ we found no associations between change in IHTAG content and change in hepatic DNL. However, we did find that the change in IHTAG was positively associated with changes in the VLDL-TAG to ApoB ratio, suggesting the greater the decrease in IHTAG, the greater the decrease in TAG-rich particles in circulation. This is in line with the previous observation that there is an overproduction of large VLDL particles with increased IHTAG content in NAFLD.^44^ Findings from in vitro work by Fisher *et al*,^45^ who used a number of hepatocyte models, suggested that the loss of large, buoyant apo-B100-containing lipoproteins in the presence of omega-3 FA is due to either: (i) omega-3 FAs preventing the particles from being assembled but without allowing the unused apoB to be secreted as higher density particles or (ii) omega-3 FA permitting the assembly of buoyant particles but then selectively inducing their destruction. Further work demonstrated that the mechanism for selective destruction was via autophagosomes.^46^ These mechanisms may, in part, explain the decrease in TAG-rich VLDL particles, we observed in the present study with omega-3 supplementation.

### Supplementation with omega-3 FAs increases plasma glucose concentrations

Studies have previously found fasting plasma glucose concentrations to increase up to 0.4 mmol/L in individuals without diabetes^20 47^ and over 1.0 mmol/L in individuals with T2D^9 11^ after omega-3 supplementation. In the present study, fasting and postprandial plasma glucose concentrations significantly increased after 8 weeks of supplementation with omega-3 FAs while having no effect on plasma insulin concentrations or markers of whole-body insulin-sensitivity (HOMA-IR). Our findings are in agreement with those of Veleba *et al*^11^ who reported an increase in fasting and postprandial plasma glucose concentrations, with no change in markers of insulin sensitivity, when metformin treated T2D individuals were supplemented with omega-3 FA (⁓2.8 g EPA+DHA/day) for 24 weeks. The authors proposed the increase in postprandial plasma glucose was due to an increase in carbohydrate and lipid availability, leading to glucose utilization being inhibited by multiple mechanisms in the Randle cycle (eg, inhibition of glucose oxidation at the level of pyruvate dehydrogenase by acetyl-CoA), reflecting peroxisome proliferator-activated receptor (PPAR)α-mediated stimulation of FA oxidation by EPA+DHA.^11^ In support of this, we observed a significant decrease in whole-body net carbohydrate oxidation rates after omega-3 supplementation and this observation along with the significant decrease in postprandial hepatic DNL, may, in part, explain the increase in postprandial glucose concentrations. Moreover, it could be speculated that the increase in fasting (and postprandial) blood glucose concentrations was a consequence of shunting glucose out of the liver due to DNL being suppressed by the omega-3 FA. Evidence is sparse for the effects of omega-3 FA on intrahepatic glucose metabolism and output, in humans in vivo.

### Omega-3 FA supplementation increases whole-body and hepatic FA oxidation but not 3OHB concentrations

In contrast to others who have assessed net fat oxidation rates using indirect calorimetry,^8 9^ we did not find an increase in the fasting state; however, after consumption of the test meal, we observed a significant increase in postprandial fat oxidation rates after omega-3 supplementation. We used stable-isotope tracer methodology to assess whole-body and hepatic ^13^CO_2_ production from recently ingested dietary fat and found both significantly increased after omega-3 supplementation. This observation may, in part, be explained by the significant decrease in postprandial hepatic DNL, which would result in cellular conditions favoring, rather than preventing, FA oxidation.^39^

Using stable-isotope methodology in our in vitro studies, we found increased FA oxidation to be evident only when omega-3 (EPA+DHA) were present. Moreover, we observed a significant increase in peroxisomal FA oxidation (as assessed by changes in media acetate levels) and ketogenesis (as measured by media 3OHB) with the presence of omega-3 FAs (EPA+DHA), despite no change or a decrease in the mRNA expression of genes involved in oxidation pathways. The observed increase in media 3OHB is in contrast to our observations in our in vivo study, and those of others^13 48^ where plasma 3OHB concentrations remained unchanged after omega-3 supplementation. A plausible explanation for the lack of change in plasma 3OHB concentrations is related to glucose sufficiency, meaning oxaloacetate was not being diverted toward gluconeogenesis and could react with acetyl-CoA to form citrate and undergo complete oxidation, rather than the acetyl-CoA being directed toward ketogenesis.^49^ Thus, the discrepancy we observed between the in vivo and in vitro data may be explained by glucose sufficiency in vivo, while in the in vitro work, cells were exposed 11 mM glucose in the media glucose, by 48 hours there was virtually no detectable glucose remaining in the media. Therefore, it is plausible that had we change the media every 24 hours rather than 48 hours, the cells would have had sufficient glucose and the increase in media 3OHB would not have been observed.

### Changes in omega-3 FA status with omega-3 FA supplementation

We found that supplementation with 1.84 g EPA+1.52 g DHA/day for 8 weeks resulted in an increase of ~224% for erythrocyte EPA but only a ~12% increase in erythrocyte DHA, with the degree of change in erythrocyte EPA achieved being inversely associated with baseline levels for EPA but there was no association between baseline and achieved erythrocyte DHA levels. Our observation, of a far larger increase in erythrocyte EPA content than erythrocyte DHA content, is in agreement with previous studies, in which EPA and DHA was given in comparable amounts^8 10 50^ or when a higher amount of DHA than EPA is consumed.^9^ The small change in erythrocyte DHA content compared with erythrocyte EPA content suggests a difference in bioavailability and/or metabolic handling. By supplementing with either EPA or DHA, Grimsgaard *et al*^51^ demonstrated that with DHA supplementation, the content of both EPA and DHA in serum phospholipids increased, and suggested that some of the observed increase in EPA was due to retroconversion of DHA to EPA. They also noted that with pure EPA supplementation, there was an increase in erythrocyte EPA content and a decrease in erythrocyte DHA content.^51^ Given these observations and the recent findings that ischemic events, including CVD, were significantly lower among individuals with elevated plasma TAG taking 4 g/day of a highly purified ethyl ester (Icosapent ethyl) than those taking a placebo,^52^ it is of interest to determine the effects of bioavailability (and mechanism of action) of both EPA and DHA. Recent evidence from the Phase III STRENGTH trial suggests that when EPA+DHA are given as carboxylic acids (*Epanova*), they provide no benefit in patients with mixed dyslipidemia who were at increased risk of CVD.^53^

### Limitations

We studied only men; therefore, it would be of interest to study the effect of omega-3 supplementation on both premenopausal and postmenopausal women to determine if there are sex-specific effects. We did not assess VLDL-TAG production or clearance rates. Omega-3 supplementation has been reported to decrease VLDL ApoB production, but not catabolism^20^ and VLDL-TAG secretion rates^47^ but has negligible effect on plasma ApoCIII concentrations, which regulate VLDL clearance.^54^ For logistical reasons, we assessed IHTAG and hepatic DNL and FA oxidation only in the omega-3 and not the placebo group; based on previous data;^17^ it is likely there would have been minimal changes in these parameters but it would be of interest to confirm this. We undertook in vitro cellular work to gain mechanistic insight; however, we cannot confirm whether both EPA and DHA were needed to have the observed effects, as the cells were exposed to both FAs; it would be of interest to determine the effects of EPA and DHA individually. Although we asked participants to maintain their habitual diet, we did not assess or control dietary intakes and thus it is plausible that the suppression in hepatic DNL resulted in a greater increase in plasma glucose concentrations in individuals who were consuming a high carbohydrate diet.

## Conclusion

**Figure 3:**
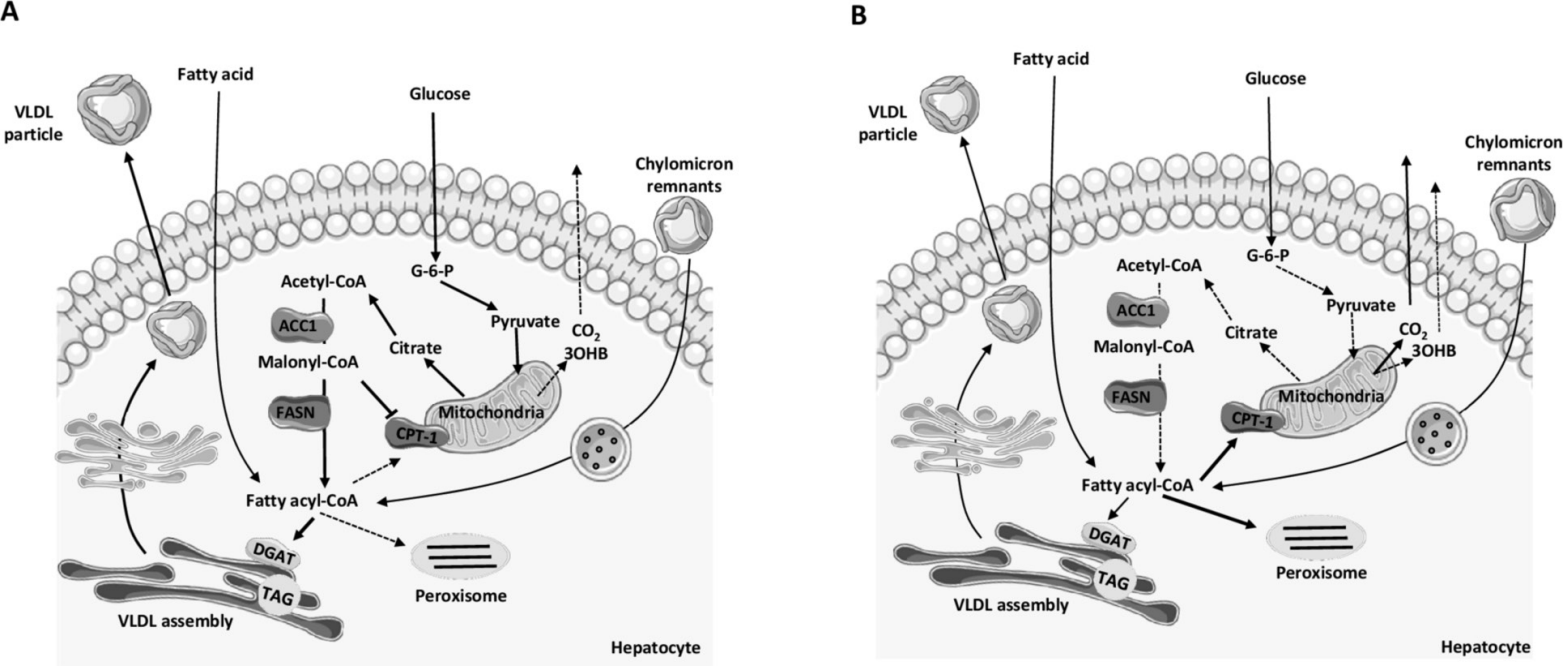
Overview of proposed effects of omega-3 FA on intrahepatic postprandial fatty acid metabolism. (A) Before omega-3 supplementation in the postprandial state, there was suppression of fat oxidation, while dietary carbohydrate was being used. Within the hepatocyte, glucose utilization would lead to an increase in acetyl-CoA which can then become a precursor for DNL, a pathway that is upregulated in the postprandial state. In DNL, acetyl-CoA is catalyzed by ACC1, to produce malonyl-CoA, an intermediate in the pathway, which is a potent inhibitor of CPT1. Inhibition of CPT1 leads to decreased fatty acyl-CoAs entering the mitochondria and peroxisomes to undergo oxidation. There is a decrease in the CO_2_ production, ketogenesis (3OHB) and acetate production. TAG production and VLDL assembly are maintained resulting in TAG-rich VLDL particles being secreted into systemic circulation. (B) After 8 weeks supplementation with the omega-3 FAs, EPA and DHA in the postprandial state fat oxidation is significantly increased and carbohydrate utilization is significantly decreased. There is no upregulation in the DNL pathway allowing fatty acyl-CoAs to enter the mitochondria and peroxisomes. There is an increase in hepatic CO_2_ production, no change in ketogenesis (3OHB) and increased acetate production. TAG production and VLDL assembly are decreased resulting in less TAG-rich VLDL particles being secreted into systemic circulation. ACC1, acetyl-CoA carboxylase; CPT-1, carnitine-palmitoyl-transferase 1; DGAT, diacylglycerol acyltransferase enzymes; DHA, docosahexaenoic acid; DNL, de novo lipogenesis; EPA, eicosapentaenoic acid; FASN, fatty acid synthase; G-6-P, glucose 6-phosphate; 3OHB, 3-hydroxybutyrate; TAG, triacylglycerol; VLDL, very low-density lipoprotein.

Taken together, our data demonstrate that omega-3 FAs (as EPA+DHA) are required to significantly alter intrahepatic DNL and FA oxidation, through potentially multiple intrahepatic mechanisms (figure 3) and the changes reported here may underpin decreases in plasma TAG concentrations and IHTAG content. However, with the notable decrease in fasting and postprandial hepatic DNL and postprandial carbohydrate oxidation, and signficant increase in fasting and postprandial plasma glucose concentrations, consideration is required for the long-term effects of attenuating hepatic DNL and potentially elevating circulating glucose concentrations on other intrahepatic and extrahepatic metabolic pathways.
