# Supplementary figures and images for: Hepatic de novo lipogenesis is suppressed and fat oxidation is increased by omega-3 fatty acids at the expense of glucose metabolism
Source: BMJ Open Diabetes Res Care. 2020 Mar 17;8(1):e000871. doi: 10.1136/bmjdrc-2019-000871 (PMC7078804; doi:10.1136/bmjdrc-2019-000871)

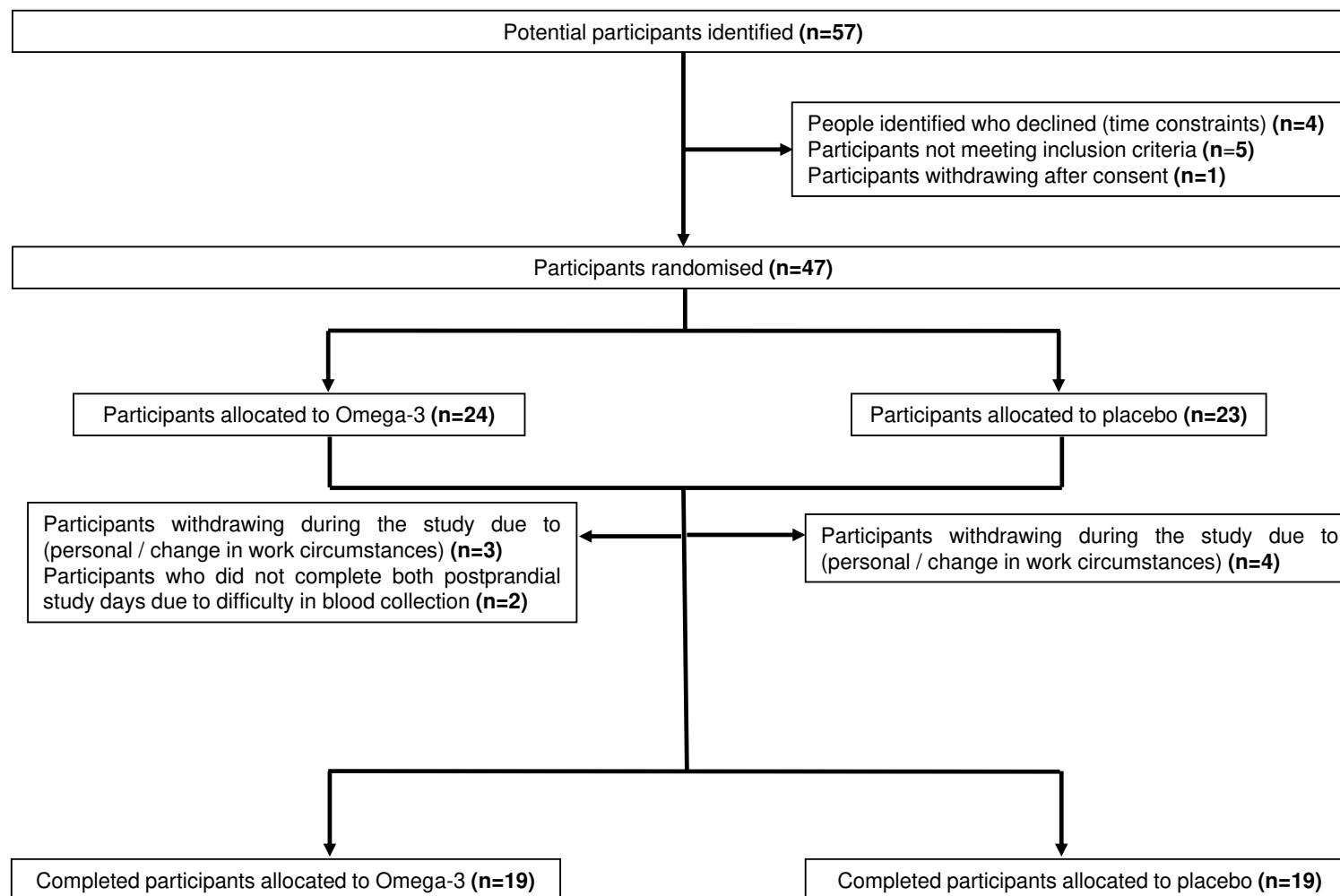

Supplementary Figure 1: Overview of participant recruitment

Supplement: Supplementary data [file bmjdrc-2019-000871supp001.pdf]
