# Supplementary material for: Hepatic de novo lipogenesis is suppressed and fat oxidation is increased by omega-3 fatty acids at the expense of glucose metabolism
Source: BMJ Open Diabetes Res Care. 2020 Mar 17;8(1):e000871. doi: 10.1136/bmjdrc-2019-000871 (PMC7078804; doi:10.1136/bmjdrc-2019-000871)
Supplement: Supplementary data [file bmjdrc-2019-000871supp002.pdf]

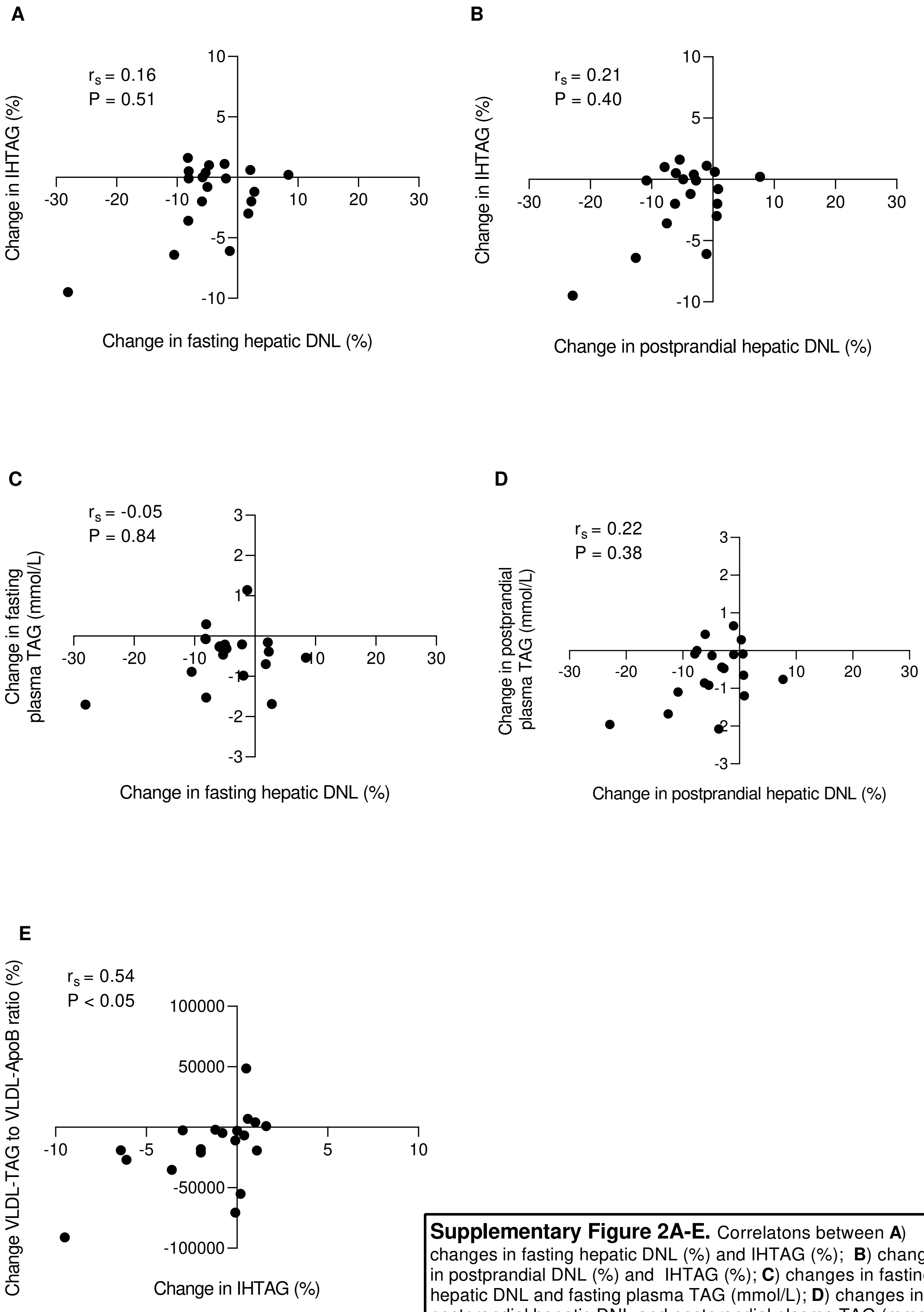

Supplementary Figure 2A-E. Correlatons between **A**) changes in fasting hepatic DNL (%) and IHTAG (%); **B**) changes in postprandial DNL (%) and IHTAG (%); **C**) changes in fasting hepatic DNL and fasting plasma TAG (mmol/L); **D**) changes in postprandial hepatic DNL and postprandial plasma TAG (mmol/L); **E**) changes in IHTAG and the VLDL-TAG to VLDL-ApoB molar ratio (%)
