# Supplementary material for: Hepatic de novo lipogenesis is suppressed and fat oxidation is increased by omega-3 fatty acids at the expense of glucose metabolism
Source: BMJ Open Diabetes Res Care. 2020 Mar 17;8(1):e000871. doi: 10.1136/bmjdrc-2019-000871 (PMC7078804; doi:10.1136/bmjdrc-2019-000871)
Supplement: Supplementary data [file bmjdrc-2019-000871supp003.pdf]

Supplementary Table 1. Relative mRNA expression in Huh7 cells.

Gene	**OPL**	**OPL+EPA+DHA**
SREBFI	1.04 ± 0.10	0.94 ± 0.16
FASN	1.02 ± 0.05	0.63 ± 0.10*
ACACA	1.05 ± 0.12	0.75 ± 0.13
DGAT2	1.06 ± 0.14	0.62 ± 0.08*
SCD	1.11 ± 0.22	0.49 ± 0.11*
PPARA	1.07 ± 0.17	0.83 ± 0.17
CPT1A	1.06 ± 0.14	0.61 ± 0.07**
CPT2	1.08 ± 0.23	0.78 ± 0.18

Data expressed as Mean ± SEM. Abbreviations: OPL, oleate, palmitate, linoleate; EPA, eicosapentaenoic acid; DHA, docosahexaenoic acid

*P<0.05, **P<0.01 OPL vs OPL+EPA+DHA

Green CJ, *et al. BMJ Open Diab Res Care* 2020; 8:e000871. doi: 10.1136/bmjdrc-2019-000871
